# E-mental Health for Elderly: Challenges and Proposals for Sustainable Integrated Psychological Interventions in Primary Care

**DOI:** 10.3389/fpsyg.2017.00118

**Published:** 2017-01-31

**Authors:** Cristina Zarbo, Agostino Brugnera, Pietro Cipresso, Ovidio Brignoli, Claudio Cricelli, Massimo Rabboni, Emi Bondi, Angelo Compare

**Affiliations:** ^1^Department of Human and Social Sciences, Human Factors and Technology in Healthcare, University of BergamoBergamo, Italy; ^2^Applied Technology for Neuro-Psychology Lab, IRCCS Istituto Auxologico ItalianoMilan, Italy; ^3^Società Italiana di Medicina Generale e delle Cure PrimarieFirenze, Italy; ^4^Department of Psychiatry, Hospital Papa Giovanni XXIIIBergamo, Italy

**Keywords:** elderly care, primary health care, technology, clinical psychology, integrative medicine

## E-mental health: improving psychology through technology

In recent years, technologies have been widely used in health setting, demonstrating their usefulness with an extensive list of diseases and medical conditions. Consequently, a new research and clinical area, called e-mental health, has emerged in the field of psychology. E-mental health refers to the use of telecommunication and information technologies to deliver mental health services (Mucic and Hilty, 2016). Psychologists began using technologies to assess or treat mental diseases as well as monitor behaviors, thoughts, feelings, and body reactions. The use of technology in psychology seems promising as it could improve the accessibility, effectiveness, quality, and affordability of mental healthcare (Lal and Adair, 2014). However, despite the several benefits of using technology in the field of psychology, numerous challenges should be taken into account in future research. In particular, there is the need to develop new integrated systems that enable healthcare providers to take care of patients using a systemic point of view in primary care settings.

Several new technologies could help psychologists and clinicians to reach this promising aim. The most used of these technologies are mobile applications, virtual reality tools, wearable sensors, ICT software, or PC/Tablet tools (e.g., email, videoconferences, virtual medical records) and web resources (e.g., online support groups, online therapies). For example, e-mental health smartphone applications could provide information to clients as well as screen, assess, monitor or treat symptoms, behaviors, thoughts, or feelings as well as provide social support (Lal and Adair, 2014; Piwek and Ellis, 2016). Furthermore, wearable sensors can collect physiological data about respiration and heart rate frequency, skin conductance, and movements (e.g., to detect falls or monitor daily activities) and give useful information about real-time body reactions. To date, the most common online therapy is the Computerized Cognitive Behavioral Treatment (CCBT) due to the several advantages it offers (e.g., it allows to improve self-management; individuals can benefit from CBT resources with less therapist involvement; Kaltenthaler et al., 2004).

Growing evidence highlights the potential benefits of new technologies for mental health support. Indeed, technologies could save time, resources and costs; increase flexibility of and accessibility to treatments; improve quality of healthcare services; and help those people who request time to reflect on themselves or therapy sessions, have difficulty expressing themselves or do not appreciate social or human contact (Lal and Adair, 2014). Computerized and technology-assisted interventions seem to be acceptable and effective in treating common mental health problems in adults, such as anxiety and depression (Andrews et al., 2010; Cuijpers et al., 2011). In particular, CCBT has shown a moderate short-term effect size for patients with depression and social phobia (Arnberg et al., 2014). Grist and Cavanagh (2013), in their systematic review and meta-analysis, found that CCBT for common mental health disorders is more effective than a waiting list or other psychotherapeutic models, even if the effect is moderated by the mean age of the patients and the specific type of control group selected. However, computerized interventions have shown low/very low effect sizes (a) for the treatment of mental disorders other than anxiety or depression, (b) for the treatment of specific populations (i.e., children/adolescents), (c) for effective improvement of cost-effectiveness and, (d) when using intervention models other than CCBT (Arnberg et al., 2014).

Finally, despite advancements in the e-mental health field, psychologists have highlighted several disadvantages that need to be considered, addressed and investigated in future research, including the lack of empathy and emotional understanding between patient and clinician as well as the lack of non-verbal communication, costs for the development and maintenance of technologies, and users' attitudinal issues.

## E-mental health for elderly in primary care

Worldwide, aging population has increased exponentially in recent decades and is expected to grow in the coming years (Cohen, 2003). Advances in medical and technological research have led to an improvement of life expectancy, even if an increasing number of older adults (a) suffer from mental disorders and disabilities and (b) experience a low quality of life or various psychological symptoms (Gallo and Lebowitz, 1999). Moreover, a large percentage of older population suffers of multimorbidity and requests polytherapy (Salive, 2013), leading to subsequent functional decline, poor quality of life, and high health care costs (Marengoni et al., 2011). Depression, anxiety disorders, alcohol abuse, and dementia seem to be the most prevalent mental disorders among the elderly (Gallo and Lebowitz, 1999). Due to the increasing prevalence, clinicians and researchers should take into account the effect of mental disorders on subjective functioning, develop preventive programs and tools and integrate mental healthcare into primary healthcare services (Gallo and Lebowitz, 1999).

“Gerontechnology”—the use of technologies in psychological settings—constitutes a promising research and clinical area. To date, technology for older adults has been used for clinical assessment and interventions, behavior monitoring, telehealth, recreational use (e.g., virtual social groups for lonely elders), information (e.g., psychoeducational modules), safety (e.g., fall detection), memory aids (e.g., cognitive training and rehabilitation), and assistance in activities of daily living (ADL) at home (Cohen-Mansfield and Biddison, 2007). In particular, smart homes and home health monitoring have been used to monitor ADL, cognitive decline, mental health as well as cardiovascular activity (Liu et al., 2016). Moreover, telehealth has been utilized for persons with severe functional disabilities and their caregivers in order to promote self-care management (Forducey et al., 2012). As Liu et al. (2016) pointed out, thus far no research has focused on the development of systems that allow for predicting disability, health-related quality of life or falls.

Evidence has suggested that new technologies for the elderly could improve quality of life, extend the length of community residence, improve physical and mental health status and delay the onset of health disorders as well as reduce family and care-giver burden (Blaschke et al., 2009). Information and communication technologies seem to improve elders' social support and psychosocial well-being (Blaschke et al., 2009). Indeed, homebound older adults who use the internet feel less isolated and, consequently, show positive outcomes (i.e., fewer depressive symptoms; White et al., 1999; Carpenter and Buday, 2007).

However, in addition to common limitations of using technology to provide healthcare, gerontechnology should address new challenges, limitations and barriers, particularly age-related issues (e.g., impaired vision, problems with manual dexterity and mobility, changes in memory, and cognition, multimorbidity and polytherapy) and training and support issues. Moreover, to date the aging population seems not yet ready for technology (Liu et al., 2016); indeed, there is a low technology-readiness level for both smart homes and home health monitoring (Liu et al., 2016). However, it has been shown that, with appropriate trainings, older adults become more interested in and can the learn skills needed to use technologies (Woodward et al., 2011). Further studies need to find new modalities to address these age-related limitations in gerontechnology even if, due to the diffusion of technology since the 1990s, even fewer elders will have difficulties in their utilization. Finally, even more studies and programs should include e-mental health in the primary care setting and follow an integrative approach that promotes collaboration among psychologists, medical practitioners and patients.

## Toward an integrative approach in primary care: the “Smart Aging” project

The efficacy and cost effectiveness of integrative medicine has been widely confirmed in studies that have not utilized technologies (Guarneri et al., 2010). However, literature lacks of projects and studies that aim to take care of older adults using an integrative point of view by means of ICT systems. Therefore, it emerges the need to develop new technologies that could overcome existing limits and barriers as well as guarantee an integrative care approach in the primary care setting. In order to overcome these limitations and reach these promising objectives, the “Smart Aging” project was developed. This is an Italian study founded by the Italian Ministry of Education, aims to develop new solutions (e.g., web platform, virtual medical records, mobile applications, wearable sensors). It aspires to improve quality of life and functioning of older adults in several aspects of life (i.e., physical activity, nutrition, psychological wellbeing, drug treatment) through new technologies.

As part of the “Smart Aging” project, University of Bergamo and involved companies (see Acknowledgment) developed three connected and integrative systems for older adults, psychologists and medical practitioners (Poster presented by Zarbo et al., 2016). In particular, we developed (a) a system called “PsiPack” which combines a mobile application with a wearable sensor for the assessment, monitoring and treating of the elder's anxious symptoms using a CCBT model; (b) a virtual medical record for psychologists that allows for real-time or weekly data-monitoring of the clients (“PsiGest”); and (c) a mobile application for medical practitioners for the weekly data-monitoring of the elder in different functioning areas (“SmartApp”).

PsiGest and SmartApp, in different modalities and times, enable the healthcare professionals to communicate with one another about their client, monitor the patient's everyday condition and weekly trends, act promptly in the case of acute negative states (e.g., high levels of arousal), manage the patient's clinical information in a more functional way and start telepresence sessions. Meanwhile, PsiPack allows for assessing baseline and post-treatment psychological conditions, monitoring everyday arousal state and worry, predicting arousal state through a specific algorithm, treating symptoms through computerized cognitive behavioral psychotherapeutic modules and sending alerts about risk conditions (e.g., highest level of arousal).

In our opinion, such integrative care approach in primary care will produce more benefits than past developed technologies as it enables patients to connect with healthcare professionals as well as psychologists and medical practitioners to share information amongst themselves. Through self-management, elders will become more aware of their emotional state and improve their own self-efficacy and sense of empowerment. Self-management has been widely shown to be a feasible and effective strategy for primary and secondary prevention as well as for empowering people with chronic and severe mental illness (Wolf, 2011). Moreover, healthcare professionals will benefit in terms of enhancing the quality of their work and ensuring prompt responses to elders' needs. In conclusion, we expect that this integrated care paradigm in primary care will reduce healthcare costs and improve quality of life among the elderly.

## Author contributions

All authors listed, have made substantial, direct and intellectual contribution to the work, and approved it for publication.

### Conflict of interest statement

The authors declare that the research was conducted in the absence of any commercial or financial relationships that could be construed as a potential conflict of interest.
